# Exploring Chinese Secondary School Students’ Acceptance of Live Video-Streamed Teaching Platforms in EFL Class: An Application of the Technology Acceptance Model

**DOI:** 10.3390/bs14070593

**Published:** 2024-07-12

**Authors:** Jinfen Xu, Qiaoling Deng

**Affiliations:** School of Foreign Languages, Huazhong University of Science and Technology, Wuhan 430074, China; xujinfen@hust.edu.cn

**Keywords:** live video-streamed teaching platforms, TAM, secondary school students, EFL

## Abstract

Live video-streamed teaching platforms are widely used in language teaching. However, how students perceive these platforms has scarcely been investigated. By adopting the Technology Acceptance Model (TAM), this study investigated Chinese secondary school students’ perceptions of the platforms (i.e., Tencent meeting, Tencent classroom and Dingtalk) being adopted in English as a foreign language (EFL) class. Gender and age differences were also investigated. Data were collected from 602 students; the results showed the following: (1) The acceptance level of all the participants was high for the five variables in TAM, i.e., perceived ease of use (PEU), perceived usefulness (PU), attitude (ATT), computer self-efficacy (CSE) and behavioral intention to use (BI), but with significant individual differences. There existed no gender differences, while age differences existed between junior high school students and those from senior high school. (2) The five variables were correlated with each other significantly. In addition, CSE, PEU, PU and ATT can predict BI in parallel. (3) The relationship between CSE and BI was mediated by PEU, PU and ATT. Also, PU had the strongest mediating effect, with PEU and ATT exerting slightly lower effects. The theoretical and practical implications are discussed at the end.

## 1. Introduction

Technology impacts our lives in myriad ways, one of which is the form of teaching and learning. In recent years, new technologies have been applied in educational settings and have shown great potential for incorporation [1], such as in foreign language teaching. Various technologies are used widely in and out of English teaching class, involving a wide range of users applying different types of technology. New technologies facilitate learning and teaching by promoting students’ learning anytime and anywhere [2]. However, if they are used without considering learners’ views and needs, learners may be resistant to the technologies and ultimately unwilling to use them [3]. Hence, in spite of the necessity and popularity of the technologies, learners, instead of technologies, should be placed at the heart of language teaching research [4]. A survey of existing literature showed that most participants in technology acceptance research in the field of language teaching and learning were teachers and students from university. Very few studies have examined secondary school students’ acceptance of technology. Therefore, this study intends to explore Chinese secondary school students’ acceptance of live video-streamed teaching platforms in English as a foreign language (EFL) class.

Before 2020, live video-streamed teaching platforms were optional. However, the outbreak of COVID-19 around the world in 2020 has made platforms such as Skype, Zoom, Dingtalk and so on compulsory rather than optional. Now, even though the COVID-19 pandemic is over, the platforms are still important complements to offline education. To the best of our knowledge, the most widely used live video-streamed teaching platforms in China are Tencent meeting, Tencent classroom and Dingtalk. They provide a virtual environment enabling real-time interactions between teachers and students similar to those in traditional class. In these platforms, a web camera and a microphone are used by teachers to give lectures and answer questions raised by students synchronously. In addition, students can interact with teachers and peers through the microphone or the chat window and download materials uploaded by teachers. Despite the convenience, challenges to learning through live video-streamed platforms still exist. Learners may find it difficult to master the features of these platforms at first. They may also be unable to keep track of the flow of the course due to the instability of the Internet, the lack of necessary hardware and the disturbance of a noisy environment. In addition, learners may engage in multitasking and become distracted by other online platforms or courses. Though live video-streamed teaching platforms are widely used in China and other countries, few studies have explored learners’ acceptance of these platforms, especially regarding secondary school students. In view of this, the paper aims to investigate Chinese secondary school students’ perceptions of Tencent meeting, Tencent classroom and Dingtalk when learning EFL. The investigation is carried out from the perspective of the Technology Acceptance Model (TAM), which will be reviewed in the next section. 

## 2. Literature Review

### 2.1. TAM

**Figure 1:**
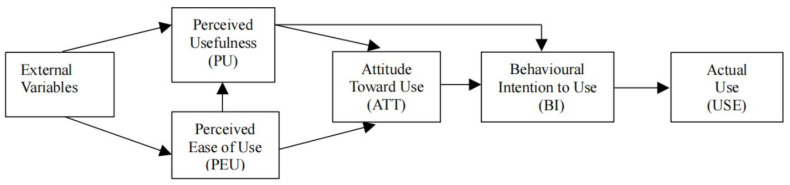
Technology Acceptance Model [6].

To examine the factors influencing technology adoption, various frameworks have been proposed, among which the TAM [5,6] is the most popular [7,8]. The model is shown in Figure 1. 

Originating from the theory of reasoned action [9], which was used to analyze the determinants of human behavior intention, TAM comprises several external variables (i.e., SN, CSE and FC), core variables (i.e., PU, PEU and ATT) and outcome variables (i.e., BI and USE). Among the core variables, perceived usefulness (PU) refers to the extent to which one person believes that adopting a certain technology would improve his or her job performance. Perceived ease of use (PEU) refers to the extent to which one person believes that using a certain technology would be effortless [6]. TAM suggested that PU, PEU and attitude toward use (ATT) directly or indirectly predict behavioral intention to use (BI) or actual use (USE) [10]. Among the external variables, computer self-efficacy (CSE), subjective norms (SN) and facilitating conditions (FC) are usually related to PU and PEU, explaining variation of users’ perceptions of the technology [11,12]. These external variables refer to factors of context and personal capabilities [13]. Contemporarily, the TAM is commonly used to examine the technology acceptance in the fields of healthcare [14,15], commerce [16,17] and tourism [18], etc.

### 2.2. TAM in Live Video-Streamed Platforms for EFL Teaching

Recently, technology adoption has been stressed in educational settings [19] and technology acceptance has become an attractive area for researchers and technology developers [20]. To investigate teachers’ and students’ acceptance of different types of technology, the TAM has been widely used across different learning contexts, such as online learning [21,22], flipped smart applications [7] and flipped learning [23], MOOCs [24,25,26], digital games [27], virtual labs [28] and virtual reality [29].

In EFL teaching, the research from the perspective of TAM involves computer-assisted language learning [30,31,32,33], automated writing evaluation (AWE) feedback [34,35], machine translation systems [36], digital textbooks [37] and mobile-assisted language learning (MALL) [38,39]. With regard to the technologies being investigated, live video-streamed teaching platforms have not received due attention. Only a few studies have explored the application of Zoom and CCtalk. Alfadda and Mahdi [40] measured 75 Saudi undergraduates’ use of Zoom in an EFL course during the pandemic of COVID-19 by correlation analysis. It was revealed that ATT, BI and USE are positively correlated. CSE was also positively correlated with PEU, PU, ATT, BI and USE. In the study of Bailey et al. [41], structural equation modeling (SEM) was adopted to analyze the TAM variables in Zoom use. Participants were 321 South Korean university students, and the research context was their EFL classes of conversational English. The results revealed that students’ PU and USE of Zoom were strongly influenced by PEU. PU predicted BI but did not influence USE. While PU predicted BI, it did not influence USE. PEU was an influential antecedent to PU, ATT and USE. Additionally, there existed a notable mediation effect through PU. Specifically, PU had mediating effect between PEU and ATT, and between PEU and BI. Wang et al. [2] also used SEM to identify factors that influenced 771 Chinese undergraduates’ adoption of CCtalk in EFL courses. The study revealed that the PEU and PU of CCtalk significantly influenced ATT. ATT and PEU significantly influenced BI. CSE significantly influenced PEU, and PEU significantly influenced PU. The three studies shed some light on learner perceptions, expectations and acceptance of live video-streamed teaching platforms in EFL teaching in different contexts. However, there are still some limitations that need to be addressed. The sample of Alfadda and Mahdi’s [40] research is small and only correlation analyses are used; thus, the effect size and the path of the variables cannot be revealed, which may influence the generalizability of the results. Bailey et al. [41] do not explore external factors such as SN, CSE and FC. Also, the sample is comparatively small. The platform CCtalk in Wang et al.’s [2] research not only performs the function of live video-streamed teaching, but also includes activities before and after class; thus, its multifunctionality may not truly or thoroughly reflect the adoption process of live video-streamed teaching platforms. Additionally, CCtalk is not a widely used live teaching platform; thus, the results of the study need to be further tested. To investigate whether there exists a difference between other platforms and across different samples, comparative research of a variety of different platforms should be implemented. To address the research gap, Tencent meeting, Tencent classroom and Dingtalk were chosen as the platforms and secondary school students as the participants of our research. Different from CCtalk or Zoom, which provide activities before or after class, these three platforms were mainly used in class, enabling synchronous interactions between teachers and students. Therefore, the environments in which the platforms were used were quite similar. Students may use the platforms in the same place and at the same time. They mainly used these platforms to listen to the lectures of teachers and answer questions raised by teachers. In TAM, SN and FC were contextual factors [35]. Considering the homogeneity of the environments in which the platforms were used, SN and FC were not investigated in this study. We chose CSE as the external variable, which may display more individual differences. 

### 2.3. Computer Self-Efficacy

Self-efficacy usually refers to a person’s subjective assessment of his or her ability to fulfill certain plans or achieve certain aspirations in the future [42]. Self-efficacy influences action choice, effort amount and persistence length when facing difficulties [43]. CSE was a strong motive influencing the action, performance and behavior of individuals in online learning [44]. The higher levels of CSE the students possessed, the more willing they were to use the technologies and the more likely they were to have a better learning performance, in addition, they were better at selecting and using different learning strategies [45]. CSE also directly affected learning engagement and self-monitoring in online learning environments [46]. Research in an education setting, from the perspective of TAM, showed that CSE positively influenced PEU [7,47], PU [47], ATT [48] and BI [49] directly or indirectly. However, Yang and Mei [50] identified no significant influence of CSE on PEU, PU and BI. The results of the research on CSE are inconsistent and the research in a language teaching context is scarce. In this vein, the effect size and the path of CSE on use of live video-streamed teaching platforms deserve further investigation.

### 2.4. Gender

Gender has been regarded as an important factor in the research of technology acceptance, and extant papers addressing gender differences have generally come to the conclusion that male students showed a greater willingness and a stronger intention to learn about and use a computer [51]. Research on gender differences based on TAM is quite inadequate. Using the TAM model, Venkatesh and Morris [52] examined differences of gender in the workplace. The results revealed that men were more likely to be influenced by PU, while women were more likely to be influenced by PEU in technology adoption decisions. As regards students, Teo and Lim [53] investigated undergraduate business administration students’ use of personal computers and found that males’ mean score of PEU was greatly higher than that of females. In a longitudinal study of undergraduates from different degrees, Padilla-Meléndez et al. [54] found that gender differences existed in BI and ATT among students using Moodle. There was also a moderator effect of gender on the use of EFL listening apps [55] and e-learning systems in compulsory blended learning environments [56]. Meanwhile, some studies found no significant gender differences when it came to using certain technologies in EFL learning, such as Zoom [40].

### 2.5. Age

Similar to gender, age is also an important variable moderating the key relationships in TAM. In spite of its importance, very little attention has been devoted to the effect of age on technology acceptance [13]. The results of empirical studies in different areas are inconsistent, and would be divided into three broad categories. Firstly, age significantly influenced the acceptance of technology. For example, in tourism, PEU was the only variable showing significant variation between groups of different ages [18]. In the educational field, the age of the respondents influenced the PU and BI pathway as well as the relationship between PEU and PU [57]. Age also significantly influenced the ATT of Vietnamese teachers towards digital teaching [58]. Secondly, some studies found that a certain age, together with other factors, had significant effects on technology acceptance. For example, there was no direct effect of PU for people who were over 35 in human resource management [59]. The results of Pratama’s [60] study indicated that variables in the TAM significantly determined m-learning acceptance, while being moderated by the effects of age, gender and location differences altogether. Thirdly, there are still some studies suggesting no evidence for the age effect. Chung et al. [61] found that there were no age differences regarding the relationship between PEU, PU and BI in online community participation. Also, age did not moderate the relationship between PU and PEU on social networking sites [62] and there existed no age effect on social influence and BI [63]. 

The literature shows that most empirical studies (83%) were carried out at universities; thus, target groups like secondary school students are potential groups in the future [1]. In the scarce research on secondary school students’ technology acceptance, it was found that CSE, PEU and PU positively affected the online learning motivation of K-12 students [64]. Additionally, Yang et al. [38] used SEM to examine Chinese secondary school students’ acceptance of MALL and found that SN and ATT toward MALL significantly predicted the BI to use it, whereas PEU and PU mediated other factors that influenced ATT. Empirical studies on secondary school students’ acceptance of live video-streamed teaching platforms are, to our best knowledge, lacking. Given the paucity of research on live video-streamed teaching platforms in secondary schools in China, and considering the lack of result generalizability in technology acceptance research [1], the current study explores secondary school students’ use of Tencent meeting, Tencent classroom and Dingtalk, aiming at extending the explanatory power of TAM to new platforms with different populations. Due to the immediacy of live video-streamed teaching platforms and the highly demanding levels of interactions between teachers, platforms and students, the results may further test the generalizability of TAM. 

## 3. Research Questions

The present study aims to investigate how Chinese secondary school students perceive live video-streamed teaching platforms that they have access to in formal language learning and the resultant learning experiences. Specifically, the study focused on three research questions (RQs):

1. What is Chinese secondary school students’ general acceptance level of live video-streamed teaching platforms in EFL class? Are there any significant differences concerning gender and age?
2. What is the relationship between core variables (i.e., PEU, PU and ATT), external variables (i.e., CSE) and outcome variables (i.e., BI)?
3. Do PEU, PU and ATT mediate the relationship between CSE and BI?

## 4. Method

### 4.1. Participants

Participants were made up of a convenience sample of 602 Chinese secondary school students from Guangzhou, Shenzhen and Wuhan. A total of 243 (40%) participants were juniors, while 359 (60%) were seniors. Participants also represented gender evenly, with 317 males (53%) and 285 females (47%). Although convenience sampling was adopted in this study, the three cities were from quite different areas in China and the schools in the three cities could represent the situation of how secondary school students used live video-streamed teaching platforms in China to a large extent. Therefore, the results may generally reflect secondary school students’ perceptions of the platforms. Because the study was carried out during the pandemic, the use of any of Tencent meeting, Tencent classroom and Dingtalk was compulsory. Additionally, the platforms were used during regular class time. Different schools may choose any of the three platforms, but in this study, the same school used the same platform.

### 4.2. Research Instruments

The research instrument employed in this study was a questionnaire, which consisted of two parts: information of demography, and measurement items validated by prior research. Demographic information concerned the information about participants’ grade, gender and school. The measurement items of technology acceptance were adapted from [13,65,66,67,68,69]. They were modified to reflect the live teaching context to better cater to our study scenarios. Tencent meeting, Tencent classroom and Dingtalk were all included in the items in order to get a more comprehensive result. In total, there were 23 items. All the items were translated into Chinese, assessed by a five-point Likert scale ranging from “strongly disagree = 1” to “strongly agree = 5”. Taking Tencent meeting as an example, indicator reliability, internal consistency reliability and convergent validity of the measurement calculated by SPSS 26.0 are shown in Table 1. As can be seen from Table 1, the results showed satisfactory indicator reliability because all the factor loadings were greater than 0.70, exceeding the benchmark of 0.50 [70]. Composite reliability (CR) and Cronbach’s alpha for each construct was greater than the recommended threshold of 0.70 [71] (ranging from 0.817 to 0.946), providing evidence of internal consistency reliability. The average variance extracted (AVE) of each construct was greater than 0.50 [72] (ranging from 0.616 to 0.797), exhibiting convergent validity of the latent constructs. The resulting factor loading, CR, Cronbach’s alpha and AVE values were all higher than the threshold values. Therefore, the questionnaire exhibited a satisfactory level of reliability and validity. We proceeded to perform descriptive analyses.

### 4.3. Data Collection and Analysis

In the present study, an online questionnaire was distributed in January 2023 via the Wen-JuanXing platform. In this period of time, the COVID-19 lockdown was not over, and live video-streamed teaching platforms were still frequently used in secondary schools. The questionnaire was given to the participants through their English teachers and all the students were informed of the research purpose, the data confidentiality and their unconditional rights of withdrawal before they filled in the questionnaire. Additionally, instructions were given on the details of the questionnaire in order to ensure that their self-reported answers could truly reflect their perceptions of the technology used in their English class. The preliminary analysis of collected data involved questionnaires with missing data and unengaged responses. The response rate was 100% (602 responses in total). 

To answer RQ 1, we conducted a descriptive analysis, normality tests and independent-sample *t* tests. To answer RQ 2, correlation analysis and multiple regressions were performed. To address RQ 3, we used Process v 4.1 (Model 6) SPSS 26.0 to calculate the size and path of mediating effects.

## 5. Results

### 5.1. Descriptive Analysis

To provide a general picture of students’ acceptance level of live video-streamed teaching platforms, descriptive statistics was conducted firstly.

As indicated by the mean scores in Table 2, in general, student participants reported high levels of CSE, PEU, PU, ATT and BI, while standard deviations and maximum and minimum scores exhibited huge individual differences between the participants. From the Skewness and kurtosis, we can see that all variables showed a normal distribution. Correlation and regression analyses were conducted subsequently.

Because the participants were mainly composed of two cohorts of students, i.e., junior and senior high school students, grade differences instead of age differences were analyzed. Independent-sample *t*-tests were conducted to explore gender and grade differences. Table 3 displays the results.

Table 3 shows there were no gender differences regarding the five variables (*p* > 0.05), while there existed significant grade differences regarding CSE, PU, ATT and BI (*p* < 0.05) and the mean scores of senior high school students were all lower than those of junior high school students. However, no gender differences existed concerning PEU (*p* > 0.05). Considering the uneven numbers of juniors and seniors, an independent-sample *t*-test with the same numbers (N = 243 for both juniors and seniors) was further carried out to see whether the difference was an artifact of the sample size. The result showed that the significance of *p* values for the five variables did not change. The grade differences among studies in regard to CSE, PU, ATT and BI were still significant. 

### 5.2. Correlation and Multiple Regressions Analyses

In order to address RQ 2, Pearson correlation analysis was conducted. The results are presented in Table 4.

In accordance with the threshold values proposed by [73], medium to large correlations were found between all the variables. To be more specific, the effect size between ATT and BI was the largest (*r* = 0.921, *p* < 0.01). Both ATT and BI were positively related to PU, and the effect size was slightly lower (*r* = 0.872, *p* < 0.01; *r* = 0.865, *p* < 0.01, respectively). With regard to the relationship between PEU and PU, ATT and BI, the correlation coefficients were above 0.75 and under 0.8 (*r* = 0.742, *p* < 0.01; *r* = 0.762, *p* < 0.01; *r* = 0.767, *p* < 0.01, respectively). Finally, the correlation coefficients between CSE and PEU and PU were above 0.7 and under 0.75, respectively (*r* = 0.705, *p* < 0.01; *r* = 0.718, *p* < 0.01), while the effect size between CSE and ATT and BI were the lowest (*r* = 0.656, *p* < 0.01; *r* = 0.687, *p* < 0.01 respectively).

To further examine the potentially predictive power of CSE, PEU, PU and ATT on BI, a multiple regression analysis (stepwise method) was conducted. Specifically, the analysis was carried out in the following four steps: (1) CSE and BI were entered as independent and dependent variables separately; (2–3) CSE was entered as the independent variable for the dependent variables of PEU, PU and ATT, respectively; and (4) CSE, PEU, PU and ATT were all entered as co-predictors for BI. The results in the regression model showed that CSE, PEU, PU and ATT all predicted the dependent variable of BI simultaneously. The results are shown in Table 5.

According to Table 5, VIF indicates no clear evidence of multicollinearity. When entered into the same regression model, the effects of external and core variable (i.e., CSE, PEU, PU and ATT) on outcome variable (i.e., BI) were still positive and significant. The effect of ATT (*β* = 0.645, *p* < 0.001) outweighed that of PEU (*β* = 0.089, *p* < 0.001) and PU (*β* = 0.190, *p* < 0.001), respectively. CSE affected BI positively and significantly, with a small effect size (*β* = 0.065, *p* < 0.01). The results indicated that when CSE, PEU, PU and ATT co-occurred, ATT took the leading role and contributed most to the level of BI, followed by PU, PEU and CSE. Additionally, the results showed that the parallel multiple mediator model fit is excellent (*R*^2^ = 0.871, F = 1011.154, *p* < 0.01) [74]. PEU, PU and ATT mediated the effect of participants’ CSE on BI collectively. Namely, CSE directly or indirectly predicted participants’ BI by means of predicting PEU, PU and ATT first.

### 5.3. Mediating Effect Analyses

**Figure 2:**
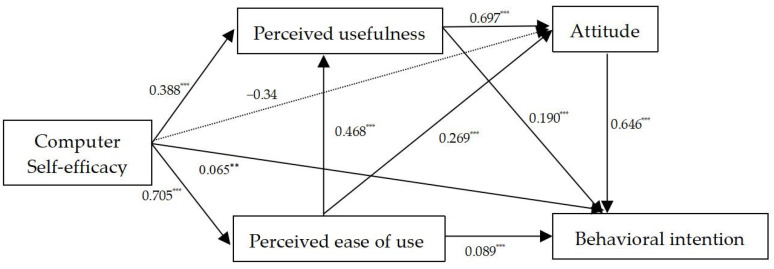
The statistical diagram of parallel multiple mediation. ** *p* < 0.01, *** *p* < 0.001.

To understand the overall relationship of the five variables and assess the mediating effect of PEU, PU and ATT, we then conducted mediation analyses by using Process v 4.1 (Model 6) SPSS 26.0, adopting the steps put forward by [75,76]. A bootstrapping analysis was performed with 5000 sub-samples. The parallel mediator model is presented in Figure 2 and the total indirect effect size and the path of PEU, PU and ATT are provided in Table 6.

Table 6 shows a significant level of total indirect effect size of 0.62, for the 95% confidence interval did not straddle zero. Combining the results with those in Figure 2, the findings are as follows: (1) The indirect effect sizes of pathways 5, 6 and 7 were comparatively high and quite close, with the respective effect sizes reaching 0.12, 0.17 and 0.15. All of them reached a significant level, for all the 95% confidence intervals did not straddle zero. Also, the mediating effect of PU between CSE and ATT was larger than that of PEU. CSE significantly predicted PEU, PU and BI (*β* = 0.705, *p* < 0.001; *β* = 0.388, *p* < 0.001; *β* = 0.065, *p* < 0.001, respectively). PEU significantly predicted PU (*β* = 0.468, *p* < 0.001) while PEU and PU positively predicted ATT (*β* = 0.269, *p* < 0.001; *β* = 0.697, *p* < 0.001). (2) The indirect effect sizes of pathways 1, 2 and 4 were much lower, with the values being 0.06, 0.07 and 0.06, in turn. In spite of the low effect sizes, they still reached a significant level. The mediating effects of PEU and PU on the relationship between CSE and BI were the same, while they were much lower than those on the relationship between CSE and ATT. PEU and PU positively predicted BI (*β* = 0.089, *p* < 0.001; *β* = 0.190, *p* < 0.001). (3) Finally, the indirect effect size of pathway 3 was the lowest and did not reach a significant level, as can be seen from the 95% confidence interval. Even though ATT positively predicted BI (*β* = 0.646, *p* < 0.001), the predicted effect of CSE on ATT was not significant (*β* = −0.034, *p* > 0.05). The indirect effect sizes of the seven pathways showed that the direct influence of CSE on BI was much lower than the effects mediated by PEU, PU and ATT. The meditating effect of PU on the relationship between CSE and ATT was the largest, followed by the co-mediating effects of PEU and PU, with the former being an antecedent to the latter.

## 6. Discussion 

### 6.1. The Profiles of Acceptance Level

The descriptive statistics revealed that Chinese secondary school students’ acceptance of live video-streamed teaching platforms was high in general. Owing to the COVID-19 pandemic, Tencent meeting, Tencent classroom and Dingtalk have been used for about three years and secondary school students have become used to operating these platforms for EFL learning. However, huge individual differences were found in their perceptions of these platforms. The possible reasons could be attributed to stability of the Internet, the online teaching environment and the teachers. If the Internet is stable, students will perceive the platforms as user-friendly; otherwise, students may feel nervous due to being afraid of making mistakes in operating the platform; thus, they may consider the platform difficult to use. For students with different characters, the introverted students may prefer the online teaching environment, in which they can interact with teachers and peers more smoothly, without the pressure of direct face-to-face contact. Additionally, if teachers have expertise in using the platforms and are able to give more instructions on how to use the platforms, students will also be inclined to use the platforms. No gender differences were found in this study. The results are consistent with the findings of [40] but do not echo those from [54,55,56]. When it comes to grade, there existed no differences in PEU between junior and senior high school students. The finding is inconsistent with the results of [18]. The possible reason is that 91% of the respondents in [18] research were between 30 and 60 years old. They may have thought that mobile apps such as QR code and GPS technology were difficult to register with and use. Meanwhile, all the participants in our study are aged between 13 to 18. Born in the Digital Age and recognized as digital natives, secondary school students heavily rely on various technologies in their daily life and studies. As a consequence, most of them have a comprehensive knowledge of how to use these modern technologies, and they tend to have no difficulty using the live video-streamed platforms, whether they are males or females. They may think the platforms are user-friendly. For CSE, PU, ATT and BI, junior and senior high school students showed significant differences. The results concur with the findings from [57,58], while being inconsistent with those from [61,62,63]. The possible reason may be that the variables investigated in the previous three studies are different from ours. For example, Uchenna and Oluchukwu [63] investigated the relationship between expectancy of performance and effort, social influence and BI. In our study, the mean scores of all the four variables for junior high school students were higher than those for senior high school students. Teachers’ different teaching approaches and students’ expectation of using the platforms may explain the result. In junior school, to arouse students’ interest and sustain their attention, teachers may resort to more vivid means, such as games or video clips. Junior students are also less exam-oriented. They may perceive no difference between learning through the platforms and in offline classrooms. For senior high school students, teachers may mainly focus on grammar or vocabulary instructions, and students are more exam-oriented, because they receive more pressure from exams. Consequently, they may think their interactions with teachers through the platforms are not as helpful as the instructions they receive in the real classroom for promoting their exam scores.

### 6.2. Relationship between CSE, PEU, PU, ATT and BI

Correlation analyses indicated that the five variables showed significant medium-to-large associations with each other, which is a popular conclusion in the research literature based on TAM [67] and concurs with the results of [2,40,41]. In the first place, CSE was found to be positively correlated with both PEU and PU. Students with high CSE are prone to perceive live video-streamed teaching platforms to be more useful and easier to use. The reason may be that they hold the belief they possess the necessary capabilities for success in using the platforms. In the second place, PEU and PU were found to be positively correlated with ATT. The reason could be that if students consider the platforms easy to use and useful, their attitudes towards using them may be more positive. Finally, ATT and BI shared the largest correlation. Students with positive ATT may be more willing to use the platforms, resulting in a higher level of BI. The regression analyses further revealed the predictive power of CSE, PEU and PU on BI. Specifically, ATT was the strongest predictor of BI, which is in line with those reported in [38,64]. PU, PEU and CSE can also predict BI directly, with the predictive power being large to small. In summary, all the external and core variables have direct effects on the outcome variable. 

### 6.3. Mediation of PEU, PU and ATT in the Relationship between CSE and BI

Even though CSE influenced BI directly, the indirect effect was much higher, as can be seen from the mediation effect analyses. The result is consistent with those of [38,41,49]. More specifically, the mediation effect of PEU and PU on the relationship between CSE and BI was almost equal, with small effect sizes in both cases. Meanwhile PEU and PU exerted high mediation effect on the relationship between CSE and ATT, with the effect size of 17 and 12 respectively. PEU could also mediate the relationship between CSE and PU. That is to say, PEU was an antecedent to PU, with the effect size of 15. Taking all the results together, the greatest predictive power of ATT on BI mainly came from the mediation effect of PEU and, especially, PU. Therefore, the effect of PU on BI is strongly supported in this study and PU is the deciding factor in acceptance of technology, including live video-streamed teaching platforms, confirming the results of many previous studies, such as the authors of [6,11,12,77], who emphasized that, in their stepwise regression analysis, nearly 60% of BI was determined by PU. 

## 7. Conclusions

By adopting TAM, this study is one of the first to examine how secondary school students perceive live video-streamed teaching platforms. The significance of the study lies in the important information added to the extant literature with regard to new platforms used by a new population in a new context. The results of the study also have some implications for theory and practice. Theoretically, the study contributes to verifing the generalizability of TAM regarding the Tencent meeting, Tencent classroom and Dingtalk platforms adopted by Chinese secondary school students. The external variable of computer self-efficacy influences the outcome variable of behavioral intention directly and indirectly. The core variables of perceived ease of use and perceived usefulness are the strongest predictors of technology acceptance, with the latter’s influence being confirmed as more robust. The results show that perceived usefulness is a key enabler of live video-streamed teaching platforms; therefore, it is the most powerful determinant for the acceptance of various technologies in education.

The findings of the study also provide practical implications for university administrators, teachers and developers of live video-streamed teaching platforms. Firstly, university administrators are advised to provide more training for language teachers on how to use technologies such as live video-streamed teaching platforms. In the digital age, technology use in teaching is necessary and unavoidable. Training on technology use can change teachers’ teaching beliefs and improve their digital literacy, which may be helpful to give more instructions to students. Secondly, the findings of the study are conducive to better understanding students’ attitudes toward the platforms and find the pathways on how to improve students’ acceptance of technology. Teachers can design tasks that can better meet students’ needs in the new learning context, such as improving interactions through online group activities by using chat window or creating breakout rooms. In addition to teaching contents, teachers should attach more importance to perceived ease of use and perceived usefulness of platforms, which are two strong predictors of technology acceptance. To be more specific, before and during using the platforms, teachers can give instructions on how to use and help students who have trouble using them, through which the perceived ease of use could be improved. At the same time, teachers can set learning goals with incremental difficulties and encourage students to exchange positive experiences in using the platforms in an attempt to improve perceived usefulness. Thirdly, the results can be used for developers’ reference to design platforms that are user-friendly and easy to use, considering that perceived ease of use is a critical factor facilitating the adoption of live video-streamed teaching platforms. At the same time, the platform should possess more functions enabling teachers and students to have more interactions; thus, students’ sense of belonging and sense of reality in synchronous online learning environment can be improved. 

As with any study, the present study also has some limitations. One limitation is that actual usage of the live video-streamed teaching platforms has not been measured. Whether a high level of behavioral intention would ultimately lead to actual use needs further investigation. The other limitation concerns the external variable being investigated. Whether facilitating conditions actually exert some influence on students’ perceptions of live video-streamed teaching platforms deserves further investigation. Also, in recent years, emotions such as enjoyment, anxiety, boredom and flow are confirmed to have significant effects on language learning in traditional classrooms. They can also be integrated into TAM to investigate whether they will have the same effects in online learning environments. In light of these considerations, measures of actual use and more external variables should be incorporated in future research.

## Figures and Tables

**Table 1:** Measurement items in the questionnaire.

Constructs	Items	Source	Factor Loading	CR	Cronbach’s α	AVE
Computer Self-Efficacy (CSE)	·Tencent meeting provides assistance when there is a language problem.	[66,67]	0.838	0.827	0.817	0.616
	·I can seek help from teachers or classmates through Tencent meeting when I have problems.		0.802			
	·Tencent meeting offers good computer self-efficacy.		0.708			
Perceived Ease of USE (PEU)	·Learning to use Tencent meeting is easy for me.	[13,65]	0.798	0.888	0.888	0.666
	·Logging in and out of Tencent meeting is fast and clear.		0.784			
	·It is easy to get materials from Tencent meeting.		0.811			
	·Overall, I believe that Tencent meeting is easy to use.		0.868			
Perceived Usefulness (PU)	·Tencent meeting helps me to learn more efficiently.	[13,65]	0.768	0.946	0.945	0.714
	·Tencent meeting improves my English academic performance.		0.834			
	·Using Tencent meeting to learn English is helpful.		0.877			
	·The audio sound and the camera in Tencent meeting add to the authenticity of English learning.		0.820			
	·Tencent meeting makes English easier to learn in formal classroom.		0.864			
	·Tencent meeting gives me more control over my learning.		0.858			
	·Tencent meeting is advantageous for learning English.		0.887			
Attitude (ATT)	·Learning English on Tencent meeting is fun.	[68,69]	0.865	0.940	0.939	0.797
	·Using Tencent meeting for learning English is a good idea.		0.882			
	·Tencent meeting is an attractive way to learn English.		0.913			
	·I like using Tencent meeting for learning.		0.910			
Behavioral Intention (BI)	·I believe Tencent meeting is useful for me as a student.	[13,65,68]	0.878	0.941	0.939	0.763
	·Tencent meeting helps me improve my English skills.		0.890			
	·I feel comfortable using Tencent meeting to improve my English.		0.841			
	·Tencent materials are useful to me for learning English classes in the future.		0.908			
	·I think Tencent meeting should be used in English.		0.848			

**Table 2:** Descriptive statistics for technology acceptance (N = 602).

Variable	Possible Range	Min.	Max.	M	SD	Skewness (*SE* = 0.1)	Kurtosis (*SE* = 0.199)
CSE	3–15	3	15	11.35	2.534	−0.766	1.603
PEU	4–20	4	20	15.27	3.290	−0.856	1.616
PU	7–35	7	35	23.48	6.542	−0.145	−0.035
ATT	4–20	4	20	14.08	3.947	−0.411	−0.068
BI	5–25	5	25	17.79	4.581	−0.469	0.496

**Table 3:** Independent-sample *t*-tests of CSE, PEU, PU, ATT and BI.

	Male	Female	*t* Value	*p* Value	Junior	Senior	*t* Value	*p* Value
	(N = 317)	(N = 285)			(N = 243)	(N = 359)		
CSE	3.78	3.79	−0.231	0.818	3.95	3.67	4.210	0.000
PEU	3.83	3.81	0.277	0.782	3.85	3.79	0.823	0.411
PU	3.36	3.35	0.118	0.906	3.48	3.26	2.935	0.003
ATT	3.47	3.57	−1.198	0.231	3.62	3.45	2.201	0.028
BI	3.54	3.58	−0.587	0.557	3.65	3.49	2.232	0.026

**Table 4:** The relationship between CSE, PEU, PU, ATT and BI.

Variable	1	2	3	4	5
1. CSE	―				
2. PEU	0.705 **	―			
3. PU	0.718 **	0.742 **	―		
4. ATT	0.656 **	0.762 **	0.872 **	―	
5. BI	0.687 **	0.767 **	0.865 **	0.921 **	―

** *p* < 0.01.

**Table 5:** Regression results for mediator model (N = 602).

Regression Equations		Fix Index			Coefficient			95.0% Confidence Interval for B		Collinearity Statistics	
Predictor	Outcome	*R*	*R* ^2^	*F*	*β*	*B*	*t*	Lower Bound	Upper Bound	Tolerance	VIF
CSE	BI	0.933	0.871	1011.154 ***	0.065	0.070	2.858 **	0.022	0.119	0.417	2.396
PEU					0.089	0.099	3.540 ***	0.044	0.154	0.343	2.917
PU					0.190	0.186	5.793 ***	0.123	0.249	0.201	4.984
ATT					0.645	0.599	20.168 ***	0.541	0.658	0.210	4.755

** *p* < 0.01, *** *p* < 0.001.

**Table 6:** Analysis of the total and indirect effects.

Pathway	Indirect Effect Size	SE	BCa 95% CI
Total indirect effect	0.62	0.04	[0.5484, 0.6892]
1.CSE→PEU→BI	0.06	0.03	[0.0107, 0.1173]
2.CSE→PU→BI	0.07	0.02	[0.0416, 0.1123]
3.CSE→ATT→BI	−0.02	0.03	[−0.0719, 0.0264]
4.CSE→PEU→PU→BI	0.06	0.01	[0.0365, 0.0913]
5.CSE→PEU→ATT→BI	0.12	0.02	[0.0813, 0.1698]
6.CSE→PU→ATT→BI	0.17	0.02	[0.1298, 0.2253]
7.CSE→PEU→PU→ATT→BI	0.15	0.02	[0.1123, 0.1873]

## Data Availability

The datasets used in the current study are available from the corresponding author on reasonable request.
